# 2H-SnS_2_ assembled with petaloid 1T@2H-MoS_2_ nanosheet heterostructures for room temperature NO_2_ gas sensing†

**DOI:** 10.1039/d4ra03194f

**Published:** 2024-08-01

**Authors:** Shraddha Hambir, Shashikant Shinde, H. M. Pathan, Som Datta Kaushik, Chandra Sekhar Rout, Shweta Jagtap

**Affiliations:** a Department of Physics, Savitribai Phule Pune University India; b MES's Department of Physics, Nowrosjee Wadia College Pune 411001 India; c UGC-DAE Consortium for Scientific Research Mumbai Centre, BARC Mumbai India; d Centre for Nano and Material Sciences, Jain (Deemed-to-be University), Jain Global Campus Ramanagaram Bangalore India; e Department of Electronic and Instrumentation Science, Savitribai Phule Pune University India shweta.jagtap@gmail.com

## Abstract

In this study, we explored the gas-sensing capabilities of MoS_2_ petaloid nanosheets in the metallic 1T phase with the commonly investigated semiconducting 2H phase. By synthesizing SnS_2_ nanoparticles and MoS_2_ petaloid nanosheets through a hydrothermal method, we achieve notable sensing performance for NO_2_ gas at room temperature (27 °C). This investigation represents a novel study, and to the best of our knowledge no, prior similar investigations have been reported in the literature for 1T@2HMoS_2_/SnS_2_ heterostructures for room temperature NO_2_ gas sensing. The formed heterostructure between SnS_2_ nanoparticles and petaloid MoS_2_ nanosheets exhibits synergistic effects, offering highly active sites for NO_2_ gas adsorption, consequently enhancing sensor response. Our sensor demonstrated a remarkable sensing response (*R*_a_/*R*_g_ = 7.49) towards 1 ppm of NO_2_, rapid response time of 54 s, baseline recovery in 345 s, good selectivity and long-term stability, underscoring its potential for practical gas-sensing applications.

## Abstract 2

In this study, we explored the gas-sensing capabilities of MoS_2_ petaloid nanosheets in the metallic 1T phase with the commonly investigated semiconducting 2H phase.

## Graphical abstract

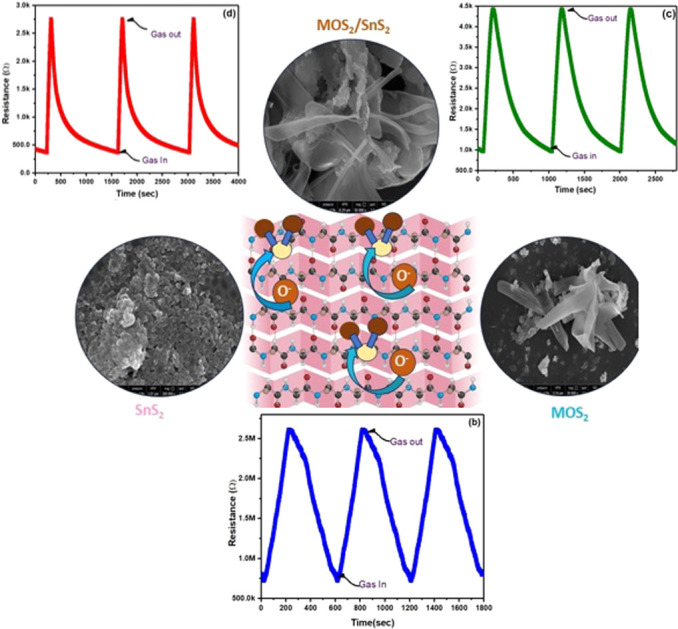

## 1 Introduction

Emission of harmful gases from various sectors such as transportation, agriculture manufacturing, construction and power generation affect both human health and the environment. To address this issue, severe regulations are being implemented globally to restrict the emission of air pollutants.^1,2^ Among these gases, NO_2_ is well known for being the most harmful gas to the entire ecosystem. If NO_2_ concentration exceeds 53 ppb, it can lead to serious health issues such as, lung disease, pulmonary irritations, chronic diseases *etc.* Hence, it is important to keep track of a number of gases in our surroundings on a regular basis^3,4^ and it is highly important to develop a cost-effective gas detection device that is efficient, precise and highly sensitive and selective.

Several methods are available for detection of toxic gases, including chemiresistive, calorimetric, optical, and electrochemical techniques.^5^ However, few methods encounter challenges like limited accessibility, higher costs, and sensitivity constraints *etc.* Resistive gas sensors have garnered considerable interest due to their compact size, ease of fabrication, straightforward operation, and cost-effectiveness in manufacturing.^6,7^ Metal oxide semiconductors (MOS) have served as effective gas sensors in the past, but their reliance on high operating temperatures led to increased power consumption. Lately, two-dimensional (2D) layer-structured materials have gained significant interest across several domains.^8^ Notably, two-dimensional semiconductor materials distinguish themselves through their extensive band gap coverage, interface free of dangling bonds, high mobility, and rapid carrier transport. A high specific surface area and rich active sites make it possible to adsorbed a large number of gas molecules, which is one of the best properties that make 2D structures advantageous in gas sensing applications.^9^ In addition, the electrical characteristics of 2D materials can be modulated by altering the number of layers.^10^ These materials undoubtedly provide a reasonably good foundation for developing high-performance gas sensors.^9^ In particular, tin disulfide (SnS_2_), a characteristic 2D layered material with weak van der Waals interactions between its layers has received significant interest.^11^ The non-solubility and non-toxicity of SnS_2_ in aqueous solutions make it a very promising material. SnS_2_ is characterised by its unique structure at the atomic layer level, which results in an abundance of available functional active sites.^12^ Being a more electronegative nanomaterial, SnS_2_ accumulates chemically adsorbed molecules on its surface, generating an electrostatic potential. The depletion region of this surface potential corresponds to the size of the entire nanostructure. This unique attribute of n-type SnS_2_ nanosheets enhances gas adsorption sites, making it a strong option for designing and making high-performance NO_2_ gas monitors.^12,13^ Moreover, the two-dimensional surface of SnS_2_ is favourable for incorporation other semiconductors, which results in the formation of well-contacted heterojunctions that improve carrier conduction.^14,15^ It has been found that the formation of heterojunctions is a highly effective method for manipulating the electronic state of the SnS_2_ surface, thereby substantially enhancing its gas sensing characteristics.^16^ Comparing heterojunction materials to individual materials, they often perform better due to their diverse morphologies and band alignments. This improvement may be linked to the excellent heterointerface, which promotes fast charge transfer. Furthermore, the curved shape of the conductive band and the valence band in a typical heterojunction structure often causes the Fermi level to attempt equilibrium, which ultimately leads in the formation of a depletion layer. This effect reduces response and recovery times by directly contributing to high conductivity.^16,17^ The construction of two-dimensional heterojunction nanomaterials has been a popular and advanced method for designing gas sensors in recent years. Similar to SnS_2_, MoS_2_ possesses a traditional layer arrangement with weak van der Waals interactions.^15^ Therefore, the development of improved heterointerfaces by employing 2D SnS_2_ and MoS_2_ nanosheets stands as a promising approach for further improving electrical capabilities. Furthermore, the metastable 1T phase exhibits superior electrical conductivity for charge transfer and higher adsorption energy to NO_*x*_ compared to the semiconducting 2H phase, suggesting its suitability for gas sensing applications. For example, MoS_2_ nanosheets coated with SnS_2_ nanoparticles (MoS_2_/SnS_2_) were developed by Jia-Bei Liu and colleagues by using the simple hydrothermal process and mechanical exfoliation method. The results of this study indicated that the SnS_2_ nanoparticles, which serve as an efficient antioxidative decoration, may increase the stability of MoS_2_ nanosheets. This provides a potential way to create high-stability NO_2_ gas sensors at ambient temperature.^18^

Considering the synergistic effects of heterostructures to boost gas sensing performance. This novel investigation explored the 1T@2H MoS_2_/SnS_2_ heterostructure by a simple hydrothermal approach to construct a two-dimensional layered 2H-SnS_2_ decorated on petaloid 1T-MoS_2_ nanosheets for room temperature NO_2_ gas sensing, marking the first instance of such research in the literature without prior similar studies. Utilizing a range of characterization techniques, comprehensive structural and functional investigations were conducted on the synthesized material. The hierarchical structure of the SnS_2_/MoS_2_ sensor proved superior to the pristine SnS_2_ sensor, exhibiting good response (*R*_g_/*R*_a_ = 7.49) to 1 ppm NO_2_ at ambient temperature. The sensor is particularly noteworthy for its excellent selectivity and consistent repeatability.

## 2 Experimental

### 2.1 Synthesis of SnS_2_ nanoparticles

SnS_2_ nanoparticles were synthesized using a one-pot hydrothermal process. The procedure involved 0.7 gm of SnCl_4_·5H_2_O, added in 30 ml of deionized water with stirring until a clear solution was formed. Subsequently, 1.2 g of CH_3_CSNH_2_ was added to the above solution, and the mixture was stirred for ten minutes. The resulting solution was then transferred to a 50 ml stainless steel autoclave lined with Teflon. Then the autoclave was maintained at 180 °C for 24 hours to facilitate the formation of SnS_2_ nanomaterials, followed by centrifugation, the precipitates subsequently subjected to overnight drying at 60 °C to obtain the final product.

### 2.2 Synthesis of 1T@2H-MoS_2_/SnS_2_ heterostructures

SnS_2_/MoS_2_ heterostructures were also formed by a hydrothermal process. To produce a uniform suspension of SnS_2_, 0.2 g of SnS_2_ was introduced to 90 ml of deionized water and ultrasonicated for 5 minutes. Later SnS_2_ solution was mixed vigorously for two hours with addition of 1.79 g of ammonium molybdate ((NH_4_)_6_Mo_7_O_24_·2H_2_O), 0.68 g of CH_3_CSNH_2_, and 0.885 g of C_19_H_42_BrN. Following that, the prepared mixture was transferred into a Teflon-lined stainless-steel autoclave with a volume of 180 ml which was then subjected to heating at 180 °C for a duration of 24 hours. After cooling to room temperature, the final product was thoroughly washed multiple times with deionized water, ethanol and dried at 60 °C. For synthesis of MoS_2_ similar procedure were carried out without addition of SnS_2_.

### 2.3 Material characterization

X-ray powder diffraction (XRD) analysis was performed using a Bruker D8 Advance instrument with Cu-Kα radiation (*λ* = 0.15406 nm). The investigation covered a range from 5° to 80° (2*θ*) to identify the crystal phase structures of the samples. The optical spectra of the material were obtained using a UV-vis spectrometer (Jasco V750). Additionally, the morphology of the synthesized material was examined through field emission scanning electron microscopy (FESEM) using a FET Nova Nano SEM 450. The current–voltage (*I*–*V*) characteristics of the semiconductor were assessed using a semiconductor parameter analysers system, (Keithley 4200A). X-ray photoelectron spectroscopy (K alpha + X-ray photo spectrometer with X-ray source – Al K). The spectra were analyzed using the XPSpeak41 software. The core peaks were deconvoluted and identified with the help of literature. The analysis of the specific surface area and pore size distribution of the synthesized material was done using the Brunauer–Emmett–Teller (BET) model and the Barrett–Joyner–Halenda (BJH) technique respectively.

### 2.4 Gas sensor fabrication and gas sensing measurement

For the gas sensing test, a gold electrode was deposited on a 1 cm by 1 cm alumina substrate through the thermal evaporation method. The device features interdigitated electrodes (IDEs) designed to facilitate gas detection. It includes two main electrodes, each 0.6 cm in length, separated by a distance of 0.4 cm. The interdigitated pattern comprises parallel fingers from each electrode, interleaved with one another. Each finger in the IDE is 0.04 cm wide, with a gap of 0.02 cm between adjacent fingers. The dimensions of the sensing film are illustrated in the ESI (Fig. S1).† Film fabrication was carried out using the drop-casting method. A suitable quantity of the synthesized material was dispersed in 5 ml of ethanol by ultrasonication for about 10 minutes. Subsequently, 10 μl of the obtained dispersion was placed at the centre of the alumina substrate, which had pre-printed interdigital gold electrodes. Gas sensing measurements were conducted using a tabletop gas sensing unit, as reported in the literature.^19,20^ We utilized a piston cylinder with a capacity of 500 ml containing a gas at a concentration of 1000 ppm. To calculate the volume required to achieve a concentration of 1 ppm in a 250 ml chamber, we applied the dilution formula:*C*_1_*V*_1_ = *C*_2_*V*_2_where, *C*_1_ = concentration of gas in cylinder = 1000 ppm, *V*_1_ = volume required for 1 ppm, *C*_2_ = concentration that we need *i.e.* 1 ppm, *V*_2_ = volume of chamber *i.e.* 250 ml and using this *V*_1_ = 0.2 ml.

Therefore, to achieve a 1 ppm concentration in a 250 ml chamber, we need 0.25 ml of gas from the cylinder. We then used syringe to collect 0.25 ml of the gas from the 1000 ppm cylinder.

## 3 Result and discussion

### 3.1 Structure and morphology

**Fig. 1:**
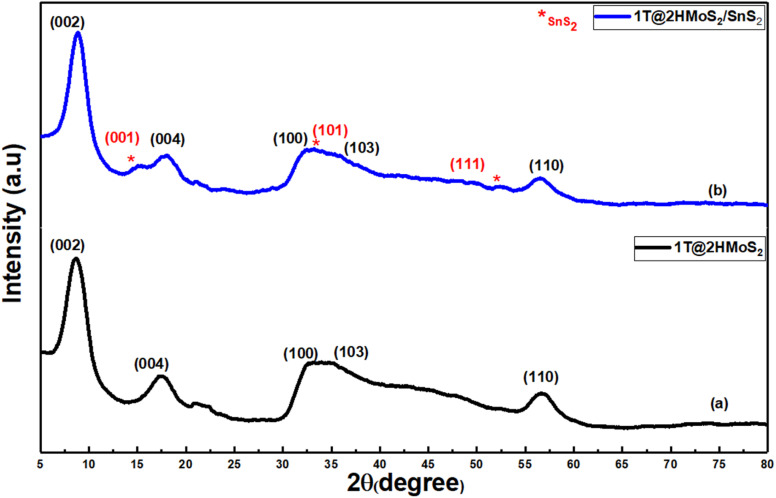
XRD diffraction patterns of (a) 1T@2H-MoS_2_, (b) 1T@2H-MoS_2_/SnS_2_ heterostructures.

Using X-ray diffraction (XRD), the crystal structure and phase purity of the synthesized materials were evaluated. Fig. 1 illustrates the XRD pattern of the as-prepared MoS_2_, and 1T@2H-MoS_2_/SnS_2_ heterostructures. Fig. 1a shows diffraction peaks at 2*θ* = 8.67°, 17.72°, 32.28°, 34.55°, and 56.51°, which correspond to the crystal planes (002), (004), (100), (103), and (110) of 1T@2H-MoS_2_ (JCPDS, No. 37-1492) further it was also observed that the peak appeared at 2*θ* = 8.67 *i.e.* (002) plane have significant displacement from 14° to 8.67°, suggesting that the interlayer spacing in 1T-MoS_2_ was greater than in 2H-MoS_2_. This shift is likely attributed to the intercalation of ammonium ions.^21^Fig. 1b shows an XRD pattern with two separate sets of peaks, one for MoS_2_ petaloid nanosheets and the other for SnS_2_ nanoparticles. XRD pattern of the as-prepared SnS_2_ nanoparticles is illustrated in Fig. S2.† Diffraction peaks at 8.89°, 17.95°, and 56.66°, which correspond to the (002), (100), and (110) planes of 1T@2H-MoS_2_ in the SnS_2_/MoS_2_ composite. In addition to this peak at 15.08°, 33.30° and 52.50° corresponds to the (001), (101) and (111) crystallographic planes of SnS_2_. Findings suggested the integration of both materials into a mixed lattice. Slight shifts in the SnS_2_ peaks towards lower angles and the MoS_2_ peaks towards higher angles indicated the presence of heterostructures qualities between MoS_2_ and SnS_2_.^22^

**Fig. 2:**
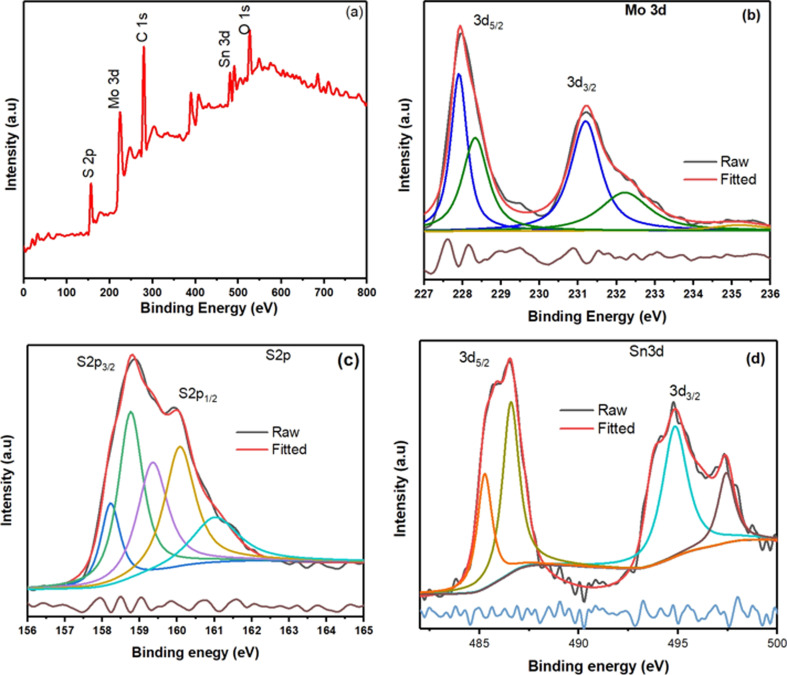
(a) XPS survey spectra of SnS_2_/1T@2H-MoS_2_ heterostructures, high resolution XPS spectra of (b) Mo 3d. (c) S 2p. (d) Sn 3d.

X-ray Photoelectron Spectroscopy (XPS) measurements were performed to determine the presence of the 1T@2H-MoS_2_ mixed phase and to examine the bonding arrangement, chemical composition, and electronic structure. The entire XPS spectrum of a SnS_2_-decorated MoS_2_ heterostructures in Fig. 2a shows Sn, Mo, S, and tiny quantities of carbon and oxygen. The presence of oxygen is a result of ambient oxygen adsorption on the composite surface. In Fig. 2b two prominent peaks, corresponding to Mo 3d_5/2_ and Mo 3d_3/2_ at around 228.9 and 231.84 eV, respectively, in the XPS spectra of the Mo 3d region for the SnS_2_/1T@2H-MoS_2_ composite show the existence of the 1T phase of MoS_2_. Two smaller 2H phase peaks with binding energies at 229.9 and 233.2 eV shift by about 1 eV to higher binding energies than 1T-MoS_2_.^23–26^ This change indicates the presence of a trace quantity of semiconducting MoS_2_ in the 2H phase. The +6-oxidation state of Mo (Mo^6+^ 3d_3/2_) is responsible for another weak peak at 236.9 eV which shows that pure MoS_2_ is partially oxidized. High-resolution S 2p spectra (Fig. 2c) show two peaks at 160.82 and 162.51 eV for S 2p_1/2_ and S 2p_3/2_ of S^2−^ from MoS_2_ one unidentified peak were observed at 160.03. The SnS_2_/MoS_2_ heterostructures is further confirmed by two distinctive peaks in the S 2p spectra of SnS_2_/MoS_2_ that are located at 160.40 and 163.47 eV, respectively and correspond to S^2−^ 2p_1/2_ and S^2−^ 2p_3/2_ from SnS_2_.^27^ In the Sn 3d region from Fig. 2d, the peaks at 487.5 eV and 496.1 eV are typical Sn(iv) peaks. Interestingly, two more peaks at 485.2 eV and 493.6 eV suggest that Sn has changed in chemical states, which may be due to SnS_2_ and MoS_2_ interactions.^28^

**Fig. 3:**
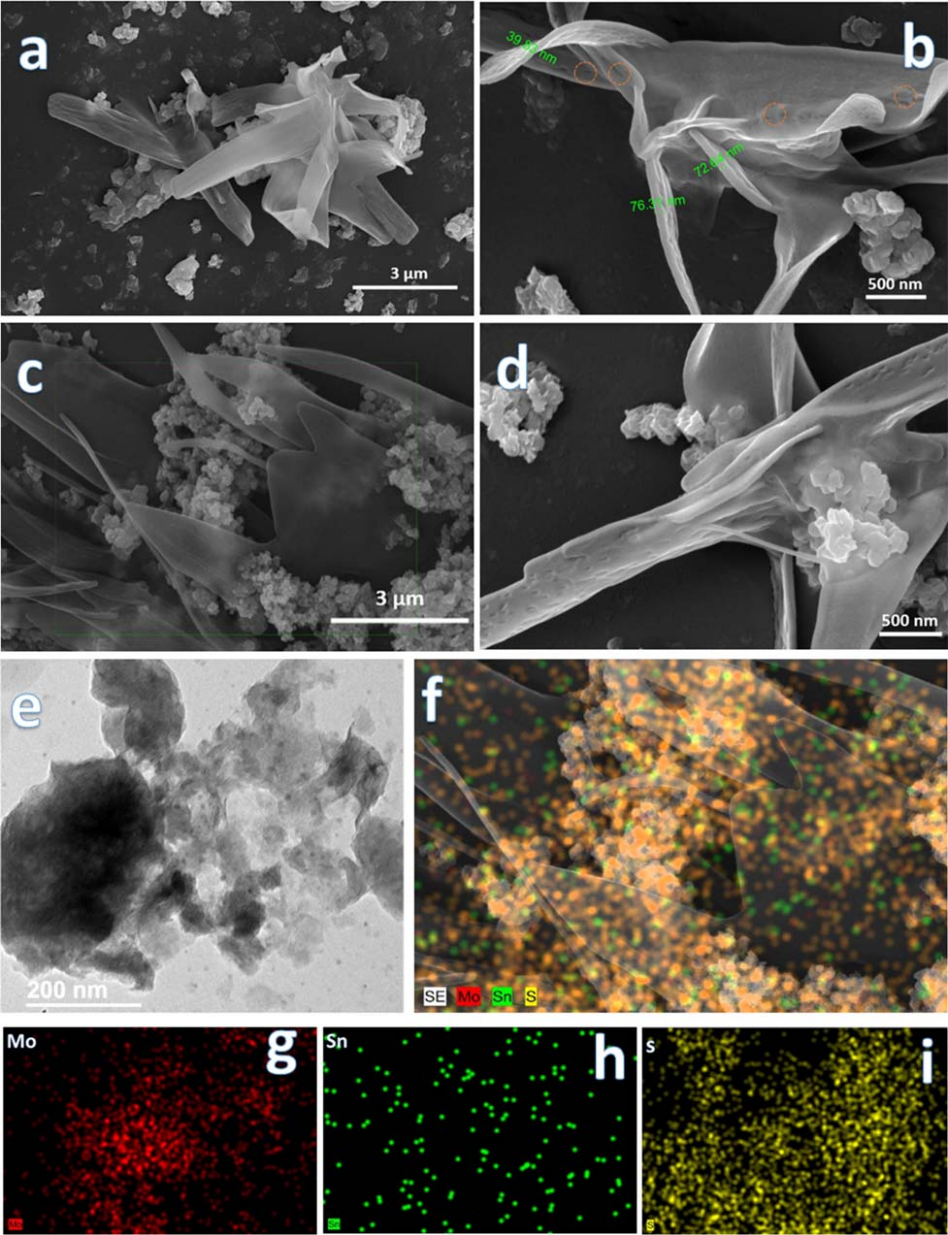
FESEM images of (a and b) 1T@2H-MoS_2_, (c and d) 1T@2H-MoS_2_/SnS_2_, (e) TEM image 1T@2H-MoS_2_/SnS_2_, (f–i) EDS mapping of 1T@2H-MoS_2_/SnS_2_ heterostructures.

The microstructures of 1T@2H-MoS_2_ and its heterojunction with SnS_2_ were investigated using Field Emission Scanning Electron Microscopy (FESEM). Fig. 3a shows pristine 1T@2H-MoS_2_, which features a combination of small nanoparticles and petaloid nanosheets ranging from 1–3 μm in length. High-resolution Fig. 3b reveals small pores on the petaloid nanosheets and width of the nanosheets is around 30–80 nm. Pure SnS_2_, illustrated in Fig. S4(a),† consists of closely packed small nanoparticles. The pristine 1T@2H-MoS_2_ also displays some nanoparticle morphology, whereas in the composite, as shown in Fig. 3c, spherical SnS_2_ particles developed on the surface of the porous 1T@2H-MoS_2_ petaloid nanosheets. Fig. 3d shows that the significant aggregation of the SnS_2_ nanoparticles, resulting in the formation of a small sheet-like structure. Fig. 3e showed TEM image of 1T@2H-MoS_2_/SnS_2_ which consist of SnS_2_ and MoS_2_ nanoparticles which are around 10 to 20 nm in size, along with MoS_2_ nanosheets. Further to confirm the presence of nanoparticles and petaloid nanosheets in 1T@2H-MoS_2_/SnS_2_, EDS analysis was also carried out as shown in Fig. 3f which clearly shows presence of sulfur (S), molybdenum (Mo), and tin (Sn) elements. Individual elemental distribution is also shown in Fig. 3g–i for Mo, Sn and S respectively.

#### 3.1.1 BET analysis

**Fig. 4:**
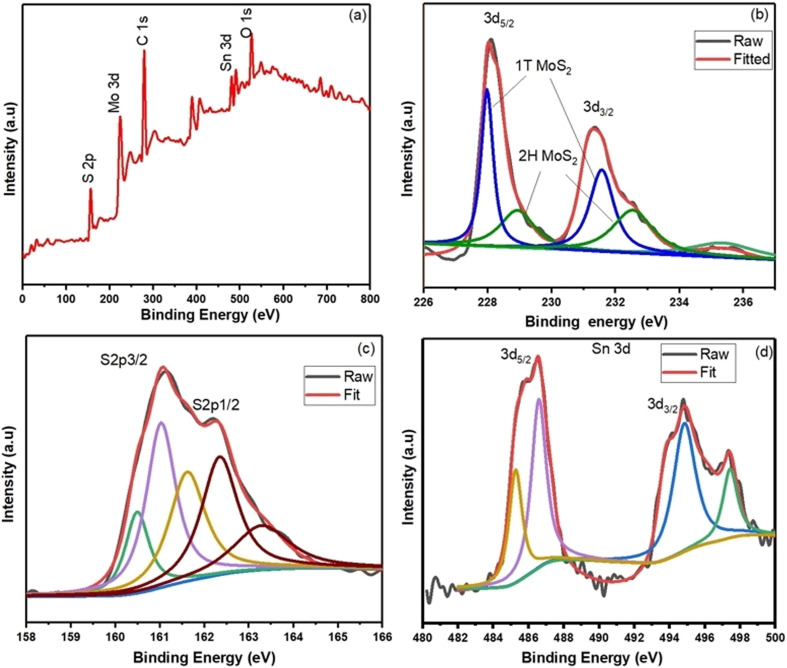
(a) N_2_ adsorption–desorption isotherms of pure 1T@2H-MoS_2_ and 1T@2H-MoS_2_/SnS_2_ heterostructures, sensor response of, (b) SnS_2_, (c) 1T@2H-MoS_2_, (d) 1T@2H-MoS_2_/SnS_2_ towards 1 ppm of NO_2_ concentration for three consecutive cycles.

The N_2_ adsorption–desorption loops for both the pure material and the heterostructures exhibit type IV adsorption isotherms (shown in Fig. 4a). The hysteresis loop (type H1 hysteresis loop) in the adsorption/desorption curve suggests the presence of mesopores in these materials^29^ with 1T@2H-MoS_2_/SnS_2_ showing lower porosity compared to 1T@2H-MoS_2_ (shown inset Fig. 4a). This difference is attributed to the introduction of SnS_2_ nanoparticles, which provide additional active sites, thereby enhancing the sensing response of the heterostructure compared to the pristine material. The calculated surface area of 1T@2H-MoS_2_ and 1T@2H-MoS_2_/SnS_2_ is 16.56 m^2^ g^−1^ and 25.45 m^2^ g^−1^ respectively. The increased surface area of 1T@2H-MoS_2_/SnS_2_ can be attributed to interconnected pores and the presence of small SnS_2_ nanospheres scattered on the surface of petaloid nanosheets. In addition, the heterostructures exhibit larger pore radius and pore volume (7.54 nm and 9.59 × 10^−2^ cm^−1^) compared to the pristine 1T@2H-MoS_2_ (3 nm and 2.48 × 10^−2^ cm^−1^). Thus, adding SnS_2_ to the MoS_2_ structure improves porosity and surface area, possibly by modifying the materials morphology and pore structure.

### 3.2 Gas sensing properties

A comprehensive investigation of the detecting capabilities of the synthesized material has been carried out, with pure SnS_2_, MoS_2_, and 1T@2H-MoS_2_/SnS_2_ heterostructures in presence NO_2_ at room temperature. Fig. 4b–d depicts the senor response of SnS_2_, MoS_2_, and 1T@2H-MoS_2_/SnS_2_ with respect to 1 ppm NO_2_ gas over three consecutive cycles. The behaviour of pure SnS_2_ is characteristic of n-type semiconductors, exhibiting an increase in resistance as shown in Fig. 4b when NO_2_ gas is introduced. Based on the gas sensing properties of 1T@2H-MoS_2_, as depicted in Fig. 4c, there is an observed increase in resistance upon exposure to NO_2_ gas, indicating that electrons are the majority charge carriers. For instance, in one study conducted by Zong *et al.* a field-effect transistor (FET) gas sensor was developed using MoS_2_ that contains a heterophase of the 1T metallic phase and the 2H semiconducting phase. The researchers also observed a decrease in conductivity when this heterophase MoS_2_ was exposed to NO_2_ gas.^30^ In the SnS_2_/1T@2H-MoS_2_ heterostructures, the interaction with NO_2_ gas is affected by the characteristics of both constituent materials. Therefore, heterostructures also exhibit a similar response, with an increase in resistance when exposed to NO_2_ as demonstrated in Fig. 4d. The response values for 1 ppm NO_2_ gas were observed as 7.49 for SnS_2_/1T@2H-MoS_2_, 5.4 for MoS_2_, and 3.49 for SnS_2_. The findings suggest that the addition of SnS_2_ nanoparticles considerably boosts the sensitivity of the 1T@2H-MoS_2_/SnS_2_ at room temperature. Furthermore, after each cycle, the resistance consistently returns to the original baseline without experiencing a significant amount of attenuation. This exceptional repeatability, which ensures constant and consistent output across many sensing cycles, is an essential feature for real-world applications.

The 1T@2H-MoS_2_/SnS_2_ heterostructures demonstrates remarkable potential for NO_2_ gas sensing with its significantly faster response and recovery times compared to individual SnS_2_ and MoS_2_. In particular, the SnS_2_/1T@2H-MoS_2_ gas sensor with response times of about 54 seconds and recovery times of about 345 seconds. This performance is higher than MoS_2_ (response time 80 s and recovery time 102 s) and the SnS_2_ sensor (response and recovery time 75 s and 91 s respectively). The faster response and recovery of the heterostructures may be attributed to a synergistic effect between SnS_2_ and MoS_2_, leading to enhanced gas adsorption and improved charge transfer dynamics.

Table 1 presents comparison between the 1T@2H-MoS_2_/SnS_2_ heterostructures and other relevant sensors, including SnS_2_-based sensors and MoS_2_-based sensors reported in the literature for NO_2_ sensing. This comparative analysis aims to provide a comprehensive evaluation of sensing performance, taking into account crucial parameters such as response, response speed, recovery time, and operating temperature. Despite some literature reports indicating sensors with higher responses, our sensor stands out due to its room temperature sensing capability, exceptional selectivity, and stability. This recognition of performance at room temperature, along with its outstanding selectivity and stability, positions our sensor as a promising candidate for widespread use, even in scenarios where other sensors may have demonstrated greater responses according to existing literature.

**Table 1:** Comparison of NO_2_ sensing performance between current data and reported data based on MoS_2_ and SnS_2_

Sensing material	NO_2_ concentration (ppm)	Working temperature (°C)	Response	Response/recovery time (s)	References
Au/SnS_2_/SnO_2_ heterojunctions	8	80	22.3	174/359.6	31
MoS_2_/rGO composite	3	160	23%	—	32
3D-MoS_2_/PbS	100	RT	25%	30/235	33
SnS_2_/MoS_2_	10	RT	6.2	3.3/25.3	34
SnO_2_@SnS_2_	0.2	RT	5.5	950/1160	35
SnS_2_/SnS	0.5	RT	2.5	375/1590	36
SnS_2_/vertical flakes	50	120	1.64	41/379	37
1T@2H-MoS_2_/SnS_2_	1	RT	7.49	54/345	This work

**Fig. 5:**
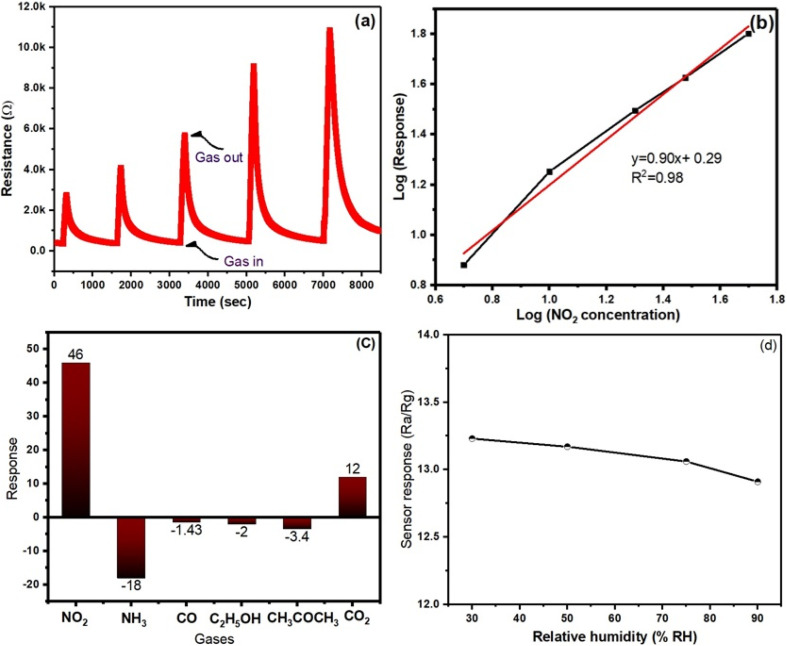
(a) Response of SnS_2_/1T@2H-MoS_2_ towards various concentration of NO_2_. (b) Sensitivity of SnS_2_/1T@2H-MoS_2_ towards various concentration of NO_2_. (c) Response of 1T@2H-MoS_2_/SnS_2_ towards 100 ppm of various gases and 10 ppm of NO_2_ at similar condition. (d) Response of 1T@2H-MoS_2_/SnS_2_ towards 5 ppm NO_2_ under different humidity level.

To get more knowledge of the NO_2_ detecting capabilities of the gas sensors, the dynamic response curves were determined at NO_2_ concentrations of 1, 5, 10, 25, and 50 ppm, respectively. As shown in Fig. 5a, the response values of the SnS_2_/MoS_2_ sensors steadily rise with increasing NO_2_ concentrations, with the 1T@2H-MoS_2_/SnS_2_ gas sensor exhibiting the greatest response value over the entire test range. Furthermore, by plotting the logarithm of sensor response on the *Y*-axis and the logarithm of gas concentration on the *X*-axis in Fig. 5b linear correlation is observed across the entire concentration range. The SnS_2_/1T@2H-MoS_2_ sensor demonstrates linear relationship, supported by an *R*-squared value of 0.987.

**Fig. 6:**
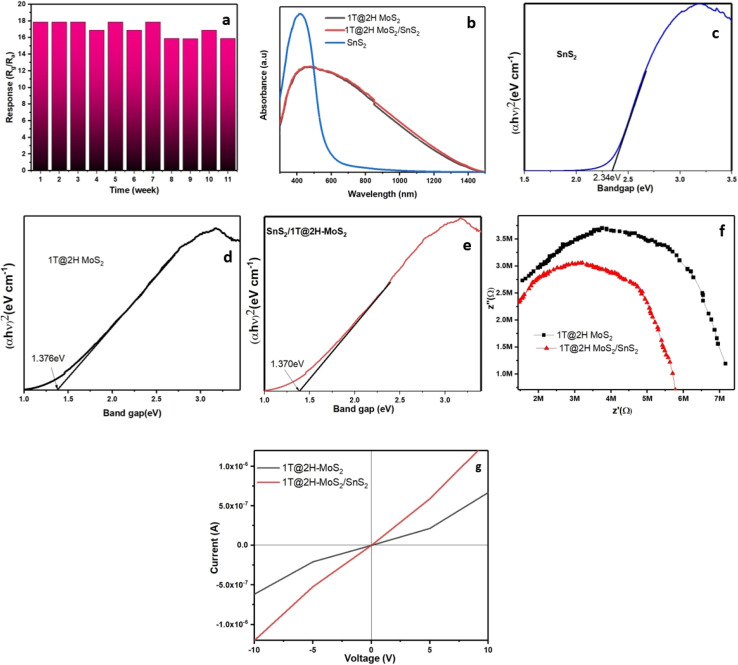
(a) 1T@2H-MoS_2_/SnS_2_ sensor stability for 12 weeks at 10 ppm NO_2_ in air, (b) UV-vis spectra of SnS_2_, 1T@2H-MoS_2_ and 1T@2H-MoS_2_/SnS_2_ Tauc plot of (c) SnS_2_, (d) 1T@2H-MoS_2_, (e) 1T@2H-MoS_2_/SnS_2_. (f) EIS plot of 1T@2H-MoS_2_ and 1T@2H-MoS_2_/SnS_2_ in presence of 10 ppm of NO_2_. (g) *I*–*V* spectra of MoS_2_ and SnS_2_/1T@2H-MoS_2_.

The selectivity of the 1T@2H-MoS_2_/SnS_2_ sensor were carried out using 1000 ppm concentration of other interfering gases, such as, ammonia (NH_3_), carbon monoxide (CO), ethanol (C_2_H_5_OH), acetone (CH_3_COCH_3_), and carbon dioxide (CO_2_) and 100 ppm of NO_2_ gas. According to the findings, which are shown in Fig. 5c, resistance increases and decreases with gas exposure are represented by positive and negative response values, respectively. The trend of selectivity for individual gas molecules is determined by their oxidation potential and electrophilicity, which are impacted by partial charge transfer and adsorption energy. Compared to other gases (CO, C_2_H_5_OH, H_2_, C_3_H_5_OH), the sensor notably shows a greater detecting response to nitrogen-based compounds (*e.g.*, NO_2_, NH_3_). This conclusion is consistent with work by Ray *et al.*,^38^ who used first-principal calculations to demonstrate that the gas adsorption energy of MoS_2_ for NO_2_ (268.6 MeV) is larger than that for NH_3_ (110.1 MeV), supporting the claim that adsorption and selectivity are related. Additionally, because of the physiosorbed paramagnetic NO_2_ molecules on the surface of SnS_2_, which provide a magnetic dipole and greater physical affinity, the device exhibits a higher sensing response to N-based substance.^39,40^ To explore the impact of relative humidity on the sensing properties of the sensor, 10 ppm NO_2_ was exposed to 1T@2H-MoS_2_/SnS_2_ under varying humidity levels (30% RH, 50% RH, 75% RH, 90% RH). The results from Fig. 5d indicate a slight decrease in the response value (13.25 ± 0.06) as the RH increases. This phenomenon can be attributed to the adsorption of water molecules, which reduces the active sites available for the target gas, consequently leading to a decline in sensor response. Therefore, it is reasonable to conclude that the influence of humidity is minimal, ensuring the reliability of the sensor in practical applications at room temperature. Furthermore, the sensor stability was examined over a twelve-week period. Results indicate that throughout this duration, the 1T@2H-MoS_2_/SnS_2_ sensor consistently shows sensing response ranging from 16.8 to 17.76 (in Fig. 6a). Therefore, development of a heterojunction between MoS_2_ and SnS_2_ considerably improves the sensors stability in air.

### 3.3 Gas sensing mechanism

Gas sensing relies on the surface interactions between the sensor material and the target gas, which alter the carrier density or the surface depletion region, consequently affecting the sensors electrical resistance. Initially, in ambient conditions, oxygen molecules adsorb onto the sensors surface, capturing electrons from the conduction band to form chemisorbed oxygen species (O_2_^−^). This process creates a wide electron depletion layer, reducing the charge carrier concentration and increasing the sensor resistance. The presence of chemisorbed oxygen enhances gas sensing response and facilitates charge transfer. When the sensor is exposed to NO_2_, a gas with electrophilic properties, it extracts additional electrons from the surface, forming adsorbed nitrite ions (NO_2_^−^). This interaction with NO_2_ modifies the concentration of adsorbed oxygen and leads to the conversion of NO_2_ into NO_3_^−^. Consequently, electrons trapped in oxygen species are released back to the conduction band, widening the depletion layer and increasing the sensors electrical resistance further.^41^

**Fig. 7:**
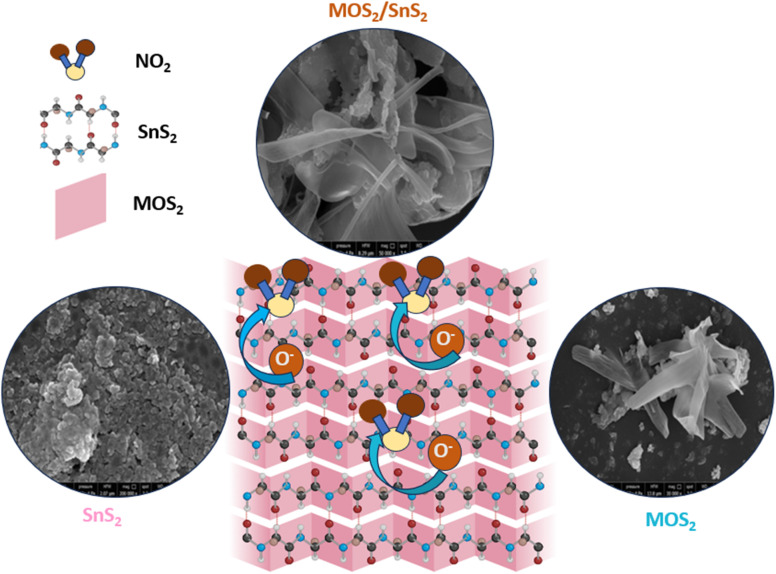
Sensing mechanism of 1T@2H-MoS_2_/SnS_2_ heterostructures.

The findings demonstrate that 1T@2H-MoS_2_/SnS_2_ heterostructures exhibit superior gas response compared to their individual counterparts. Enhanced sensor response due to the combined action of the geometric (as shown in Fig. 7) and electronic aspects. The geometric aspect results in SnS_2_ nanoparticles on the MoS_2_ surface with more exposed active sites, while the electronic aspect creates a heterostructures at the interface. According to the UV-vis spectra, pure SnS_2_ shows a high absorption, especially in the ultraviolet region, with a notable decrease in absorption beyond 400 nm. SnS_2_ coated 1T@-2HMoS_2_, on the other hand, exhibits absorption from UV to near-infrared light regions. Which indicates that it is capable of absorbing superior amounts of ultraviolet and visible light. Because of the presence of the 1T-MoS_2_ metallic phase in the composite, there is a possibility that the increased absorption in the visible light range is associated with plasmon resonance absorption (illustrate in Fig. 6b). In case of semiconductor band gap is affected by a number of parameters, such as grain size, doping, and composition. A very small change was observed by the introduction of SnS_2_ in 1T@2H-MoS_2_/SnS_2_ than the pristine 1T@2H-MoS_2_. The relationships between (*αhv*)^2^ and photon energy are shown in Fig. 6c–e. The bandgap of SnS_2_ and 1T@2H-MoS_2_/SnS_2_ are 2.34 eV and 1.36 eV respectively. The lower band gap observed in the composite material than the pristine materials. It is often simpler for electrons to go from the valence band to the conduction band when the band gap is narrower. This promotes the growth of chemisorbed oxygen on the surface of SnS_2_/1T@2H-MoS_2_, ultimately resulting in an elevated reaction rate.^42^

To further understand the interface charge transfers on the sensor surface, Electrochemical impedance spectroscopy (EIS) with equivalent circuit (illustrate in Fig. S5 and S6†) was employed in presence of 10 ppm of NO_2_ gas. Typically, impedance spectra exhibit semi-circles at low-frequency regions (illustrate in Fig. 6e), reflecting the surface charge characteristics of the sensor material. Notably, 1T@2H-MoS_2_/SnS_2_ displays a smaller semicircle indicating lower electron transfer resistance compared to other devices. These results underscore the superior gas sensing properties of 1T@2H-MoS_2_/SnS_2_ as a sensing material. Consequently, the findings suggest that the resistance and charge transfer resistance of pure 1T@2H-MoS_2_ (5.48 MΩ) is higher than those of 1T@2H-MoS_2_/SnS_2_ (3.72 MΩ). In addition to this *I*–*V* characteristics of the synthesized materials obtained at room temperature under air are illustrated in Fig. 6g. Both forward and reverse-biased regimes of the corresponding *I*–*V* curves exhibit linearity, suggesting the ohmic nature of the contact. The average electrical resistances in air are 15 MΩ, and 6 MΩ of 1T@2H-MoS_2_ and 1T@2H-MoS_2_/SnS_2_ respectively. However, a recent study by L. Liu *et al.*^34^ observed lower conductivity (resulting in higher resistance) in the case of SnS_2_/MoS_2_-II (SMS-II) compared to the pure SnS_2_ and MoS_2_ in air at room temperature. Contrary to these findings, we observed higher conductivity in the case of the 1T@2H-MoS_2_/SnS_2_ heterostructure compared to the pristine materials. This enhanced conductivity in the heterostructure may be attributed to two main factors: firstly, the introduction of SnS_2_ into the MoS_2_ lattice can act as a dopant, introducing additional charge carriers. Secondly, the presence of SnS_2_ can modify the band structure of MoS_2_, leading to alterations in the electronic properties of the heterostructure. Furthermore, the addition of SnS_2_ to the MoS_2_ structure improves porosity and surface area, contributing to the enhanced sensing response. Moreover, SnS_2_ and MoS_2_ possessing work functions of 4.2–4.5 eV and 5.2–5.4 eV respectively, heterojunctions form between them. Electron flow occurs from SnS_2_ to MoS_2_ until their Fermi levels are balanced, creating an electron depletion layer on MoS_2_ and bending the energy band of SnS_2_.^34^ This leads to a change in electrical resistance in 1T@2H-MoS_2_/SnS_2_ heterostructures. Upon exposed to NO_2_ gas at the optimal operating temperature, trapped electrons are released back to the conduction band of 1T@2H-MoS_2_/SnS_2_ heterostructures due to the reaction between adsorbed O_2_ species and NO_2_ molecules. Consequently, the electrical resistance of 1T@2H-MoS_2_/SnS_2_ heterostructures significantly decreases, resulting in an enhanced gas sensing response.

### 3.4 Conclusion

In summary, this study successfully synthesized SnS_2_ nanoparticles decorated on petaloid 1T@2H-MoS_2_ nanosheets using a simple one-pot hydrothermal method, achieving room temperature NO_2_ gas sensing for the first time. This heterostructures demonstrated effective performance as a high-quality NO_2_ sensing than the pristine materials. The 1T@2H-MoS_2_/SnS_2_ sensor exhibited remarkable response characteristics (*R*_a_/*R*_g_ = 46 at 100 ppm) including a quick response time and efficient recovery. The petaloid nanosheet morphology of 1T@2H-MoS_2_ within the heterostructure significantly enhances its performance in gas sensing applications. This structure provides a high surface-to-volume ratio, which is key in increasing the number of active sites available for gas interactions. This feature is important in improving both the sensitivity and selectivity of the heterostructure toward specific gases. In addition to this outstanding gas sensing capabilities can be attributed to the formation of heterojunctions between the 1T@2H-MoS_2_ and SnS_2_ which provide abundant electron transfer channels between the constituent materials. By exploring ternary composite materials, controlling defects in the surface by functionalization or doping, and tailoring the band gap will give new opportunities for enhancing gas adsorption and reaction efficiency.

## Data availability

All data generated or analyzed during this study are included in this article.

## Author contributions

Shraddha Hambir: conceptualization of this study, methodology, formal analysis, writing original draft and investigation. Shashikant Shinde: conceptualization and XPS analysis. Shweta Jagtap: conceptualization, supervision, validation, project administration and writing-review and editing and funding acquisition. Chandra Sekhar Rout: supervision, writing-review and editing. H. M. Pathan: supervision, validation, project administration and writing-review and editing. Som Datta Kaushik: supervision, validation, project administration and writing-review and editing.

## Conflicts of interest

The authors declare no conflict of interest.

## Supplementary Material

RA-014-D4RA03194F-s001
