# Supplementary material for: 2H-SnS2 assembled with petaloid 1T@2H-MoS2 nanosheet heterostructures for room temperature NO2 gas sensing
Source: RSC Adv. 2024 Aug 1;14(33):24130–40. doi: 10.1039/d4ra03194f (PMC11293367; doi:10.1039/d4ra03194f)
Supplement: RA-014-D4RA03194F-s001 [file RA-014-D4RA03194F-s001.pdf]

Supplementary Information (SI) for RSC Advances.
This journal is © The Royal Society of Chemistry 2024

Supplementary Information

2H-SnS_2 assembled with petaloid 1T@2H-MoS_2 nanosheets heterostructures for room temperature NO_2 gas sensing

Shraddha Hambir[a,e], Shashikant Shinde[b], H. M. Pathan[a], Som Datta Kaushik[c], Chandra Sekhar Rout[d] Shweta Jagtap[e*]

[a]Department of Physics, Savitribai Phule Pune University, India

[b]MES's Department of Physics, Nowrosjee Wadia College, Pune 411001, India

[c]UGC-DAE Consortium for Scientific Research Mumbai Centre, BARC, Mumbai, India

[d]Centre for Nano and Material Sciences, Jain (Deemed-to-be University), Jain Global Campus, Ramanagaram, Bangalore, India

[e]Department of Electronic and Instrumentation Science, Savitribai Phule Pune University, India

Corresponding author: - *shweta.jagtap@gmail.com

2. Experimental

2.4 Gas sensor fabrication and gas sensing measurement

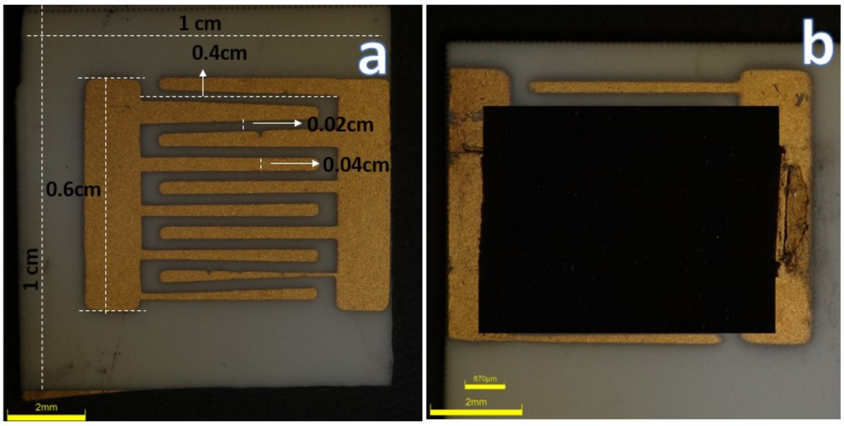

Figure S1. Optical images of Sensing device a) bare electrode, b) with material deposition

X-ray Diffraction

Figure S1 shows diffraction peaks at 15.92°, 29.12°, 33.03°, 47.48°, 50.62°, and 53.34°, which correspond to the (001), (100), (101), (102), (110), and (111) planes of 2T-type hexagonal berndtite SnS_2 (JCPDS, No. 23-0677)[1]

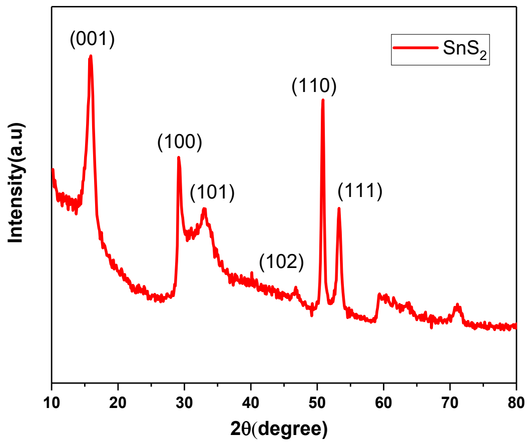

Figure S2. XRD pattern of SnS_2

Raman analysis –

The presence of 2H-SnS_2 was confirmed using non-destructive Raman spectroscopy in figure S2. Both 2H-SnS_2 and 4H-SnS_2 nanosheets exhibit a characteristic peak around 315 cm^{-1}. SnS_2 samples display an intense peak at approximately 313 cm^{-1}, attributed to the A_{1g} mode, which involves the out-of-plane stretching of sulfur atoms in SnS_2. Additionally, for the 4H-SnS_2 crystal, two weak E-mode peaks are observed at 200 cm^{-1} and 214 cm^{-1}. In contrast, the 2H-SnS_2 crystal features a weak E_g mode at 205 cm^{-1}, which differentiates it from the 4H-SnS_2. The presence of several additional modes in the Raman spectrum of 4H-SnS_2 further supports the confirmation of the 2H polytype SnS_2 formation[2–4].

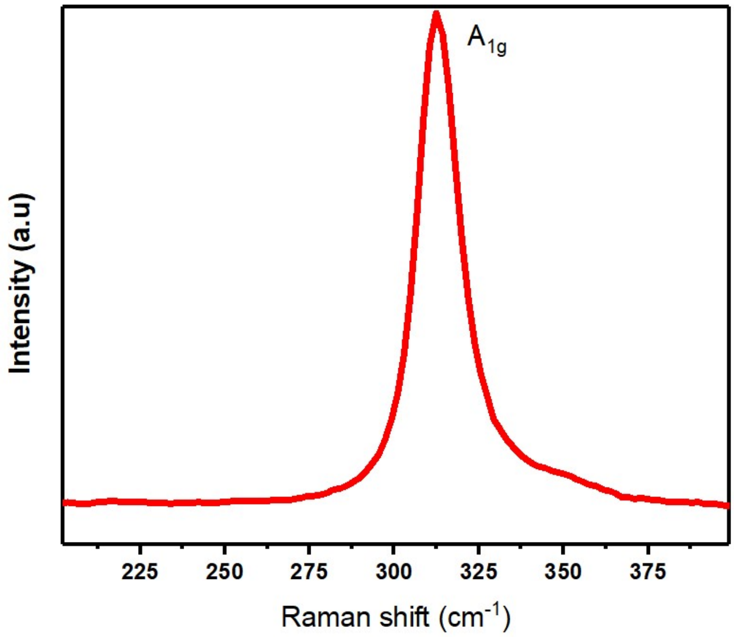

Figure S3. Raman spectra of 2H-SnS_2

FESEM

The FESEM image of pristine SnS_2 in figure S4 showed highly agglomerated nanoparticles they are densely packed and compact.

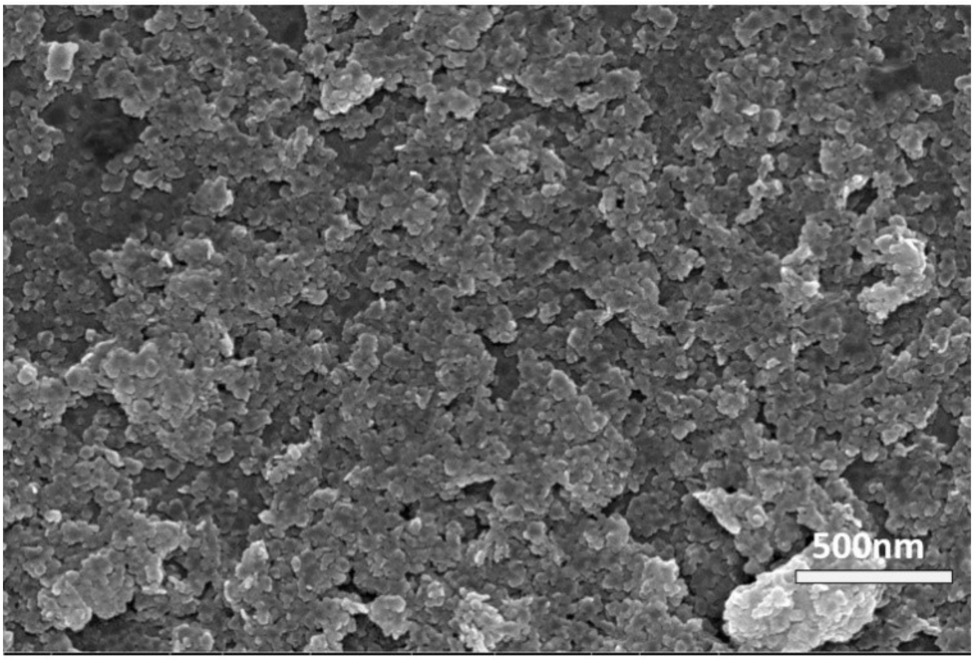

Figure S4. FESEM images of pure SnS_2

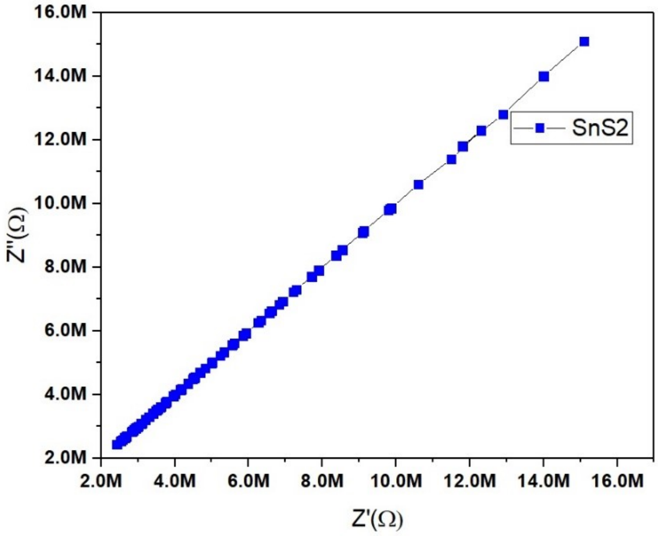

Figure S5. EIS spectra of SnS_2

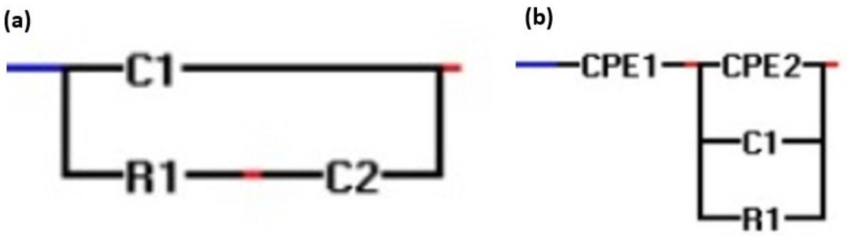

Figure S6. Equivalent Circuit for a) 1T@2H-MoS_2 and 1T@2H-MoS_2/SnS_2 and b) SnS_2

References:

1 Z. Zhang, J. Huang, M. Zhang, Q. Yuan and B. Dong, *Appl Catal B*, 2015, **163**, 298–305.

2 Y. Huang, E. Sutter, J. T. Sadowski, M. Cotlet, O. L. A. Monti, D. A. Racke, M. R. Neupane, D. Wickramaratne, R. K. Lake, B. A. Parkinson and P. Sutter, *ACS Nano*, 2014, **8**, 10743–10755.

3 T. Sriv, K. Kim and H. Cheong, 2018, **8**, 10194.

4 L. Wang, X. Li, C. Pei, C. Wei, J. Dai, … X. H.-C. C. and undefined 2022, *ElsevierL Wang, X Li, C Pei, C Wei, J Dai, X Huang, H LiChinese Chemical Letters, 2022•Elsevier*.
